# Supplementary material for: Soil Sample Analysis of Bacillus anthracis Contaminated Animal Burial Sites
Source: Microorganisms. 2024 Sep 25;12(10):1944. doi: 10.3390/microorganisms12101944 (PMC11509789; doi:10.3390/microorganisms12101944)
Supplement: Supplementary file 1 [file microorganisms-12-01944-s001.zip › File S2.pdf]

Soil sample analysis of *Bacillus anthracis* contaminated animal burial sites

SUPPLEMENTARY DATA (2)

Table S1: Excel table with data on the Kars soil samples (see Supplementary Data file 1).

Table S2: SNP genotype data for ten *B. anthracis* strains isolated from soil samples examined in this study reported by Khmaladze *et al*. [13].

District	Village	Site No.	Sample position	Type of contamination	Contamination Date	Sampling Date	Spore concentration (CFU / g soil)	Topography of site	Genotypes according to: Khmaladze *et al.* [13]			
Kars Center	γ	E	1	Surface	October 2012	October 2012	1009	Residential	A3.a	A.Br.Aus94	A.Br.013/015	A.Br.029/030
Kars Center	δ	F	4	Surface	August 2010	May 2014	124000000	Pasture	A3.a	A.Br.Aus94	A.Br.013/015	A.Br.026/027
Kars Center	ζ	H	1	Surface	July 2012	July 2012	808000	Pasture	A3.a	A.Br.Aus94	A.Br.013/015	A.Br.026/027
Kars Center	η	I	1	Animal burial	August 2013	October 2013	132	Wasteland	A3.a	A.Br.Aus94	A.Br.013/015	A.Br.026/027
Kars Center	θ	J	1	Surface	August 2013	August 2013	19860	Pasture	A3.a	A.Br.Aus94	A.Br.013/015	A.Br.026/027
Arpaçay	ι	K	1	Animal burial	2012	July 2012	1363	Cart track	A3.a	A.Br.Aus94	A.Br.013/015	A.Br.026/027
Digor	μ	N	1	Animal burial	July 2009	July 2009	52	Pasture	A3.a	A.Br.Aus94	A.Br.013/015	A.Br.028/029
Selim	ξ	R	1	Animal burial	2006-2007	June 2012	378.5	Wasteland	A3.a	A.Br.Aus94	A.Br.013/015	A.Br.028/029
Selim	ξ	R	1	Animal burial	2006-2007	May 2014	1330	Wasteland	A3.a	A.Br.Aus94	A.Br.013/015	A.Br.028/029
Selim	ξ	R	2	Animal burial	2006-2007	May 2013	133	Wasteland	A3.a	A.Br.Aus94	A.Br.013/015	A.Br.026/027

Table S3: Mixed model analysis of spore counts at different sampling positions at contaminated sites.

The output of a mixed model analysis of spore counts in samples taken from different sampling positions at known contaminated sites is shown. Fixed effects were also calculated and the role of position was found to be a probable predictor (p<0.001). Position 10 was taken as the base value. Coefficients, standard error and 95% upper and lower confidence intervals are listed.

Mixed model analysis of spore counts at different sampling positions

Model Term	Coefficient	Std. Error	t	Sig.	95% Confidence Interval	
					Lower	Upper
Intercept	0.282	.9487	.297	0.767	−1.588	2.152
Position=1	1.627	.9171	1.774	0.077	−0.180	3.434
Position=2	1.385	.9307	1.488	0.138	−0.449	3.219
Position=3	1.148	.9397	1.222	0.223	−0.704	3.000
Position=4	3.181	.9371	3.395	0.001	1.334	5.028
Position=5	1.286	.9663	1.331	0.185	−0.619	3.190
Position=6	4.061	1.0111	4.017	< 0.001	2.069	6.054
Position=7	3.258	1.0103	3.225	0.001	1.267	5.249
Position=8	0.646	1.2431	.520	0.604	−1.803	3.096
Position=9	1.787	1.1061	1.615	0.108	−0.393	3.967
Position=10	0	.	.	.	.	.
Minimum days passed	0.000	0.0001	−0.965	0.335	0.000	0.000

Table S4: Estimated means of spore counts at different sampling positions at known contaminated sites.

Estimated means and upper and lower 95% confidence intervals for spore counts at sampling positions 1 to 10 are listed. These are shown graphically in Figure 1B.

Spore Count Estimates

Position	Mean	Std. Error	95% Confidence Interval	
			Lower	Upper
1.00	1.723	.264	1.203	2.243
2.00	1.481	.339	.814	2.148
3.00	1.244	.383	.489	1.999
4.00	3.277	.407	2.474	4.079
5.00	1.381	.457	.481	2.282
6.00	4.157	.587	3.000	5.314
7.00	3.354	.595	2.181	4.527
8.00	.742	.938	-1.106	2.591
9.00	1.882	.746	.412	3.353
10.00	.096	.928	-1.733	1.924

Continuous predictors are fixed at the following values:

Min_days_passed=1686.1078

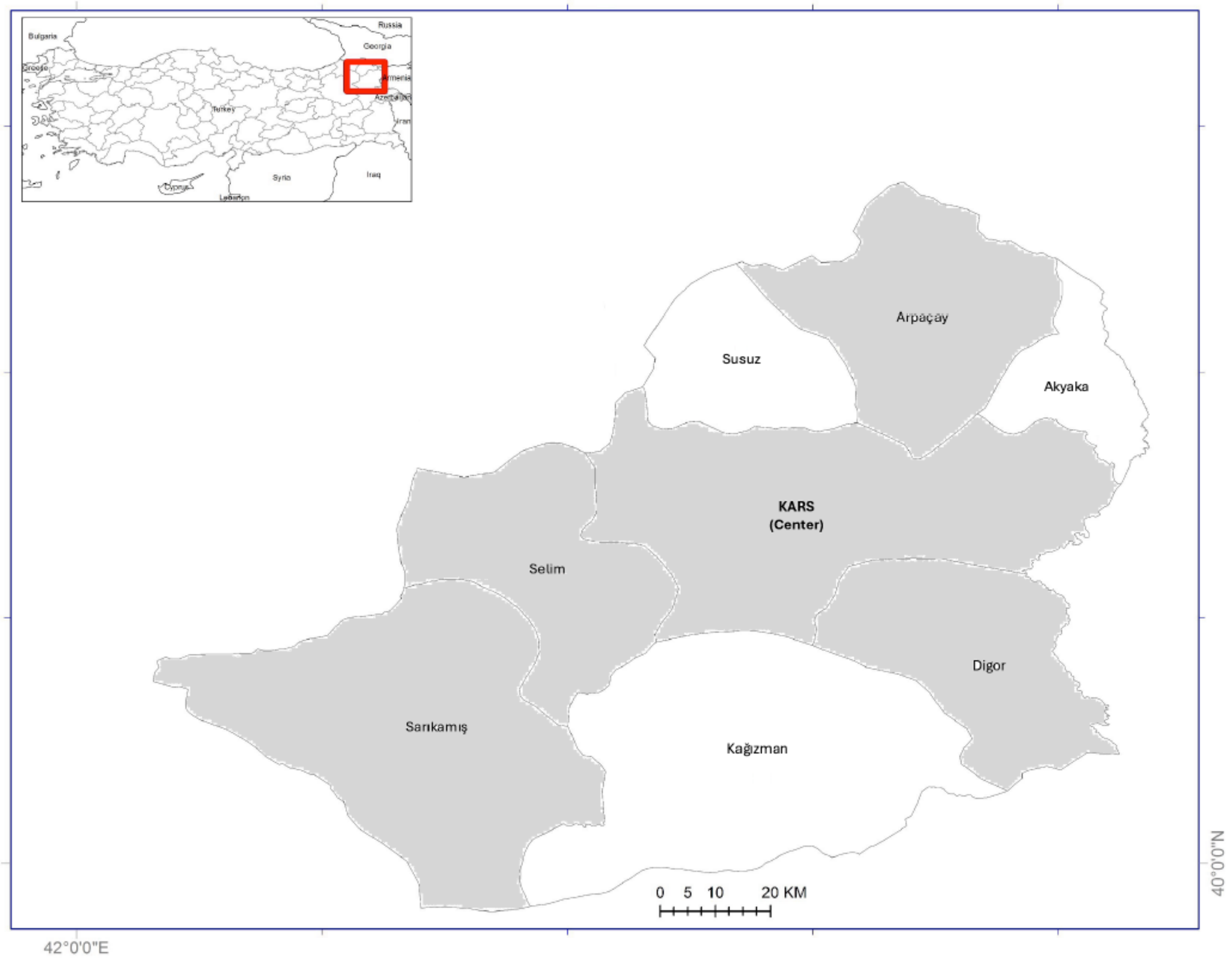

Figure S1: Map of Kars Province in eastern Türkiye showing its eight administrative districts.

B. anthracis spore contaminated sites sampled for this study were situated in or near villages within the five disticts coloured grey.

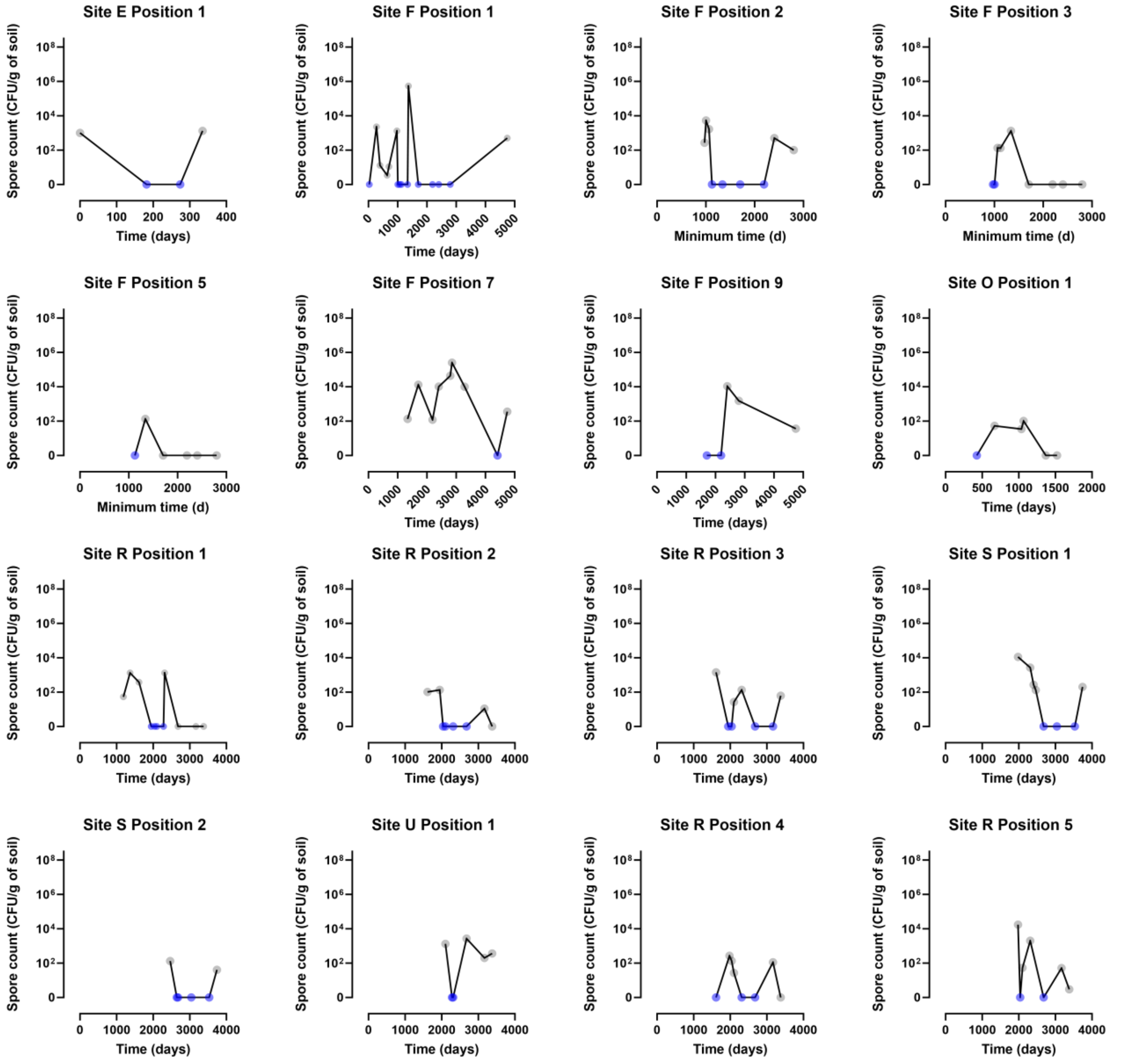

Figure S2: Plots of *B. anthracis* spore counts relative to time since contamination, at sampling positions where spores were not isolated at one time but were detected subsequently at the same position.

Blue data points indicate samples in which no spores were detected. At seven positions (Site E[1], Site F[2], Site R[1], Site R[2], Site S[1], Site S[2] and Site U[1]) samples fluctuated from being positive through negative to positive for *B. anthracis* spores. At a further four positions (Site F[1], Site R[3], Site R[4] and Site R[5]), samples fluctuated repeatedly between negative and positive at different time points.
